# Supplementary material for: Long-Chain Polyunsaturated Fatty Acids, Homocysteine at Birth and Fatty Acid Desaturase Gene Cluster Polymorphisms Are Associated with Children’s Processing Speed up to Age 9 Years
Source: Nutrients. 2020 Dec 31;13(1):131. doi: 10.3390/nu13010131 (PMC7824114; doi:10.3390/nu13010131)
Supplement: Supplementary file 1 [file nutrients-13-00131-s001.pdf]

nutrients

MDPI

Supplemental Methods

Long-chain polyunsaturated fatty acids, homocysteine at birth and fatty acid desaturase gene cluster polymorphisms are associated with children´s processing speed up to age 9 years.

SNP selection and genotyping

Genotyping of SNPs (single-nucleotide polymorphisms) from the *FADS* gene cluster was performed with the iPLEX method (Sequenom, San Diego, CA, USA) (see main text for more information). Initially, seventeen SNPs from the *FADS1* (rs174548, rs174556 and rs174561), *FADS2* (rs174570, rs174574, rs174575, rs174576, rs174578, rs174579, rs174602, rs498793, rs968567, rs2727271 and rs3834458) and *FADS3* (rs174448, rs174449 and rs174455) gene cluster from the NUHEAL mothers were genotyped **(Supplemental Table 1)**. Specific SNPs, including rs174556, rs174561, rs3834458, rs174548, rs174574 and rs174578, were selected due to its relation with FA levels [1,2], while 11 remaining SNPs were included according to its localization in *FADS* gene cluster on chromosome 11. Based on the linkage disequilibrium data from Lewontin´s D\` (D\` > 0.9) and pairwise-squared correlations r^2 ($r^2 > 0.7$), 4 SNPs out of the initial 17 SNPs (*FADS1* rs174556 and rs174561, *FADS2* rs174575 and rs3834458) were considered for further analyses **(Supplemental Figure 1)**. Hardy-Weinberg *(H-W)* equilibrium for the genotypes was calculated using the statistical software R (Version 3.2.2, package "genetics"). Deviations from the H-W equilibrium were analyzed by Fisher's exact test **(Supplemental Table 2)**, where *FADS1* rs174561 was discarded due to its low significance ($p = 0.016$). To test the linkage disequilibrium, Lewontin's D' and pairwise squared correlations r^2 were also performed **(Supplemental Table 3)**. Finally, we have selected the *FADS1* rs174556 as a representative of the block and were included in our following analysis.

Supplemental Table 1. Characteristics of the 17 analysed variants in *FADS 1/2/3* gene cluster of NUHEAL population

SNP	Possible Functional Region	Alleles (Major/Minor) and Frequency 0/1	Number (%) of subjects with Genotype			*p*
			0	1	2	
**rs174548**	*FADS 1*	G/C (0.71/0.28)	75 (0.54)	50 (0.36)	15 (0.10)	0.148
**rs174556**	*FADS 1*	G/A (0.74/0.26)	80 (0.57)	47 (0.34)	13 (0.09)	0.128
**rs174561**	*FADS 1*	A/G (0.76/0.24)	81 (0.61)	38 (0.29)	13 (0.10)	**0.016**
**rs174570**	*FADS 2*	C/T (0.89/0.11)	112 (0.79)	27 (0.19)	2 (0.01)	0.672
**rs174574**	*FADS 2*	C/A (0.65/0.35)	65 (0.46)	56 (0.39)	21 (0.15)	0.138
**rs174575**	*FADS 2*	C/G (0.71/0.29)	74 (0.52)	53 (0.37)	15 (0.11)	0.230
**rs174576**	*FADS 2*	C/A (0.67/0.33)	67 (0.48)	54 (0.38)	20 (0.14)	0.128
**rs174578**	*FADS 2*	T/A (0.67/0.33)	67 (0.48)	54 (0.38)	20 (0.14)	0.128
**rs174579**	*FADS 2*	C/T (0.78/0.22)	92 (0.64)	42 (0.29)	10 (0.07)	0.135
**rs174602**	*FADS 2*	T/C (0.75/0.25)	78 (0.55)	59 (0.41)	6 (0.04)	0.266
**rs498793**	*FADS 2*	C/T (0.52/0.48)	37 (0.26)	74 (0.52	32 (0.22)	0.739
**rs968567**	*FADS 2*	C/T (0.85/0.15)	103 (0.72)	37 (0.26)	3 (0.02)	1
**rs2727271**	*FADS 2*	A/T (0.91/0.09)	116 (0.82)	24 (0.0.17)	1 (0.01)	1
**rs3834458**	*FADS 2*	T/Z (0.68/0.32)	68 (0.49	55 (0.39)	17 (0.12)	0.249
**rs174448**	*FADS 3*	T/C (0.59/0.41)	50 (0.35)	71 (0.49)	23 (0.16)	0.864
**rs174449**	*FADS 3*	T/C (0.60/0.40)	50 (0.35)	70 (0.49)	22 (0.16)	0.862
**rs174455**	*FADS 3*	T/C (0.600.40)	50 (0.35)	69 (0.49)	23 (0.16)	1

p: level of significance according to Fisher exact test; Bold: p <0.05

0= Homozygous major, 1= Heterozygous, 2 = Homozygous minor; *FADS*: Fatty Acid Desaturase.

Supplemental Figure 1. Pairwise LD measured in D′ **(a)** and r² **(b)** for selected maternal *FADS* SNPs

a) Pairwise LD in D′ **b) Pairwise LD in *r*²**

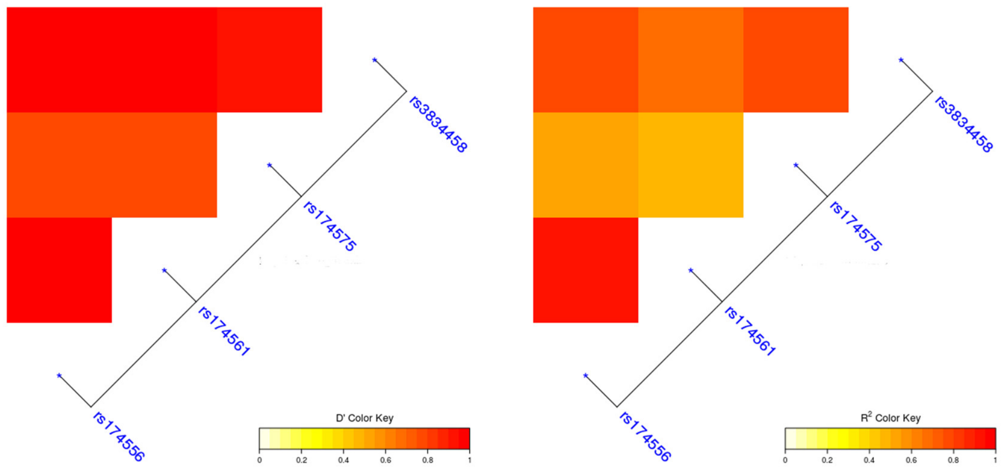

Supplemental Table 2. Characteristics of the 4 selected variants in *FADS 1/2* gene from the initial 17 SNPs analyzed in the NUHEAL population

SNP	Supplementation group	Alleles (Major/Minor) and Frequency 0/1	Number (%) of subjects with Genotype			*p*
			0	1	2	
***FADS1* rs174556**		G/A (0.74/0.26)	80 (0.57)	47 (0.34)	13 (0.09)	0.127
	FO	G/A (0.67/0.33)	22(0.41)	13(0.52)	4(0.07)	0.671
	5-MTHF	G/A (0.66/0.34)	12 (0.38)	12 (0.56)	5 (0.06)	0.591
	FO+5-MTFH	G/A (0.73/0.27)	22 (0.68)	10 (0.27)	-	0.538
	Placebo	G/A (0.79/0.21)	24(0.62)	12(0.35)	4 (0.04)	1
***FADS1* rs174561**		A/G (0.76/0.24)	81 (0.61)	38 (0.29)	13 (0.10)	**0.016**
	FO	A/G (0.68/0.32)	22 (0.43)	12 (0.50)	4 (0.07)	0.671
	5-MTHF	A/G (0.67/0.13)	12(0.40)	10 (0.53)	5 (0.07)	0.616
	FO+5-MTFH	A/G (0.75/0.25)	23 (0.68)	7 (0.32)	0	1
	Placebo	A/G (0.80/0.20)	24 (0.64)	9(0.32)	4 (0.04)	1
***FADS2* rs174575**		C/G (0.71/0.29)	74 (0.52)	53 (0.37)	15 (0.11)	0.230
	FO	C/G (0.71/0.29)	21 (0.48)	14 (0.45)	5 (0.07)	0.274
	5-MTHF	C/G (0.75/0.25)	12 (0.45)	14 (0.45)	3(0.10)	1
	FO+5-MTFH	C/G (0.73/0.27)	20 (0.50)	11 (0.45)	3 (0.05)	0.385
	Placebo	C/G (0.75/0.25)	21 (0.54)	14 (0.42)	4 (0.04)	0.448
***FADS2* rs3834458**		T/Z (0.68/0.32)	68 (0.49	55 (0.39)	17 (0.12)	0.249
	FO	T/Z (0.64/0.34)	18 (0.38)	15(0.55)	2 (0.07)	0.413
	5-MTHF	T/Z (0.66/0.34)	11 (0.38)	13 (0.56)	5 (0.06)	0.591
	FO+5-MTFH	T/Z (0.75/0.25)	19 (0.55)	2 (0.41)	2 (0.05)	1
	Placebo	T/Z (0.73/0.27)	20 (0.50)	15 (0.46)	5 (0.04)	0.631

p: level of significance obtained from Hardy-Weinberg Equilibrium Test; Bold: p <0.05

0= Homozygous major; 1= Heterozygous; 2=Homozygous minor; 5-MTHF: 5-Methyltetrahydrofolate; *FADS*: Fatty Acid Desaturase; FO: Fish-oil.

Supplemental Table 3. Pairwise linkage disequilibrium analysis in maternal selected *FADS* SNPs

		rs174561	rs174575	rs3834458			rs174561	rs174575	rs3834458			rs174561	rs174575	rs3834458
**rs174556**	***n***	119	125	125	**rs174561**	***n***	---------	119	118	**rs174575**	***n***	---------	---------	125
	D	0.184	0.146	0.184		***D***	---------	0.137	0.170		***D***	---------	---------	0.187
	D′	0.999	0.782	0.999		***D′***	---------	0.779	0.999		***D′***	---------	---------	0.919
	r	0.959	0.719	0.867		***r***	---------	0.687	0.832		***r***	---------	---------	0.868
	***X*²**	219.29	129.26	187.96		***X*²**	---------	112.43	163.31		***X*²**	---------	---------	188.51
	p	**<0.001**	**<0.001**	**<0.001**		***p***	---------	**<0.001**	**<0.001**		***p***	---------	---------	**<0.001**

p: level of significance obtained from correlation analysis. Bold: $p < 0.05$

$$1-\beta = \Phi\left(z - z_{1-\alpha/2}\right) + \Phi\left(-z - z_{1-\alpha/2}\right) \quad , \quad z = \frac{\mu_A - \mu_B}{\sigma\sqrt{\frac{1}{n_A} + \frac{1}{n_B}}}$$

D: coefficient of linkage disequilibrium; *D´*: normalizing D; *n*: number of cases; *r*: correlation coefficient; X^2: Chi-square

Statistical Analysis

Power calculation was performed for the present analysis using the following formula [3]:

Formula Power calculation →

where: σ is standard deviation; ϕ is the standard Normal distribution function; ϕ^{-1} is the standard Normal quantile function; α is Type I error and β is Type II error, meaning 1-β is power.

Supplementary Table 4 shows the results obtained, where z indicates power calculation, considering α= 0.05 and β= 0.2 (80%).

Supplemental Table 4. Power calculation obtained for processing speed evaluation in NUHEAL children

Speed processing tasks at 7.5 years	Pooled Intervention group (FO+5MTHF+MIX)	Placebo group	z

SDMT – hits	22.87 ± 6.22	24.66 ± 6.38	0.80
CCTT-1 (sc)	86.12 ± 41.12	99.88 ± 50.77	0.80
STROOP Test – hits -1 (Word-Reading)	60.13 ± 14.85	59.34 ± 16.41	0.80
STROOP Test – hits -2 (Color-Naming)	41.30 ± 8.12	41.95 ± 8.41	0.80

Values expressed as mean ± standard deviation (SD).

5-MTHF: 5-Methyltetrahydrofolate; ***CCTT***: Children Color Trails test; FO: Fish-oil; MIX: FO + 5-MTHF; ***SDMT***: Symbol Digit Modalities test; z= power calculation

Interestingly, power calculation for genetic study was also calculated using the package “PowerEQTL v0.1.3” (R software). For this purpose, we considered an one-way unbalanced ANOVA with different ranges of sample size (50, 100, our-case 143, 200, 250 and 300), α value of 0.05 and 17 analyzed SNPs in *FADS* 1/2/3 gene cluster. As show in **Supplemental Figure 2 and Supplemental Table 5**, a statistical power of 90% was obtained for analyzed SNPs, except for rs174570 and rs2727271.

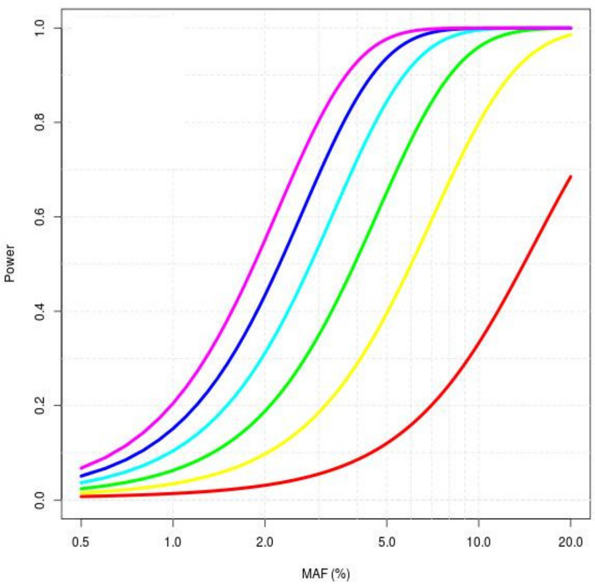

Supplemental Figure 2. Power estimation, α=0.05, SNPs=17 (one-way unbalanced ANOVA). X-axis represents the power calculation, and y-axis shows the percentage of minor allele frequency. Red: sample size (*n*) of 50 subjects; yellow: *n* = 100; green: sample size in the present study (*n*= 143); light blue: *n*= 200; dark blue: sample size of 250 subjects; violet: *n*= 300.

Supplemental Table 5. Power calculation obtained for genetic study in NUHEAL children

SNPs	Possible Functional Region	Alleles Frequency (Major/Minor)	Power Calculation
**rs174548**	*FADS1*	G/C (0.73/0.27)	>0.90
**rs174556**	*FADS1*	G/A (0.74/0.26)	>0.90
**rs174561**	*FADS1*	A/G (0.75/0.25)	>0.90
**rs174570**	*FADS2*	C/T (0.90/0.10)	>0.80
**rs174574**	*FADS2*	C/A (0.68/0.32)	>0.90
**rs174575**	*FADS2*	C/G (0.73/0.27)	>0.90
**rs174576**	*FADS2*	C/A (0.68/0.32)	>0.90
**rs174578**	*FADS2*	T/A (0.67/0.33)	>0.90
**rs174579**	*FADS2*	C/T (0.81/0.19)	>0.90
**rs174602**	*FADS2*	T/C (0.77/0.23)	>0.90
**rs498793**	*FADS2*	C/T (0.55/0.45)	>0.90
**rs968567**	*FADS2*	C/T (0.83/0.17)	>0.90
**rs2727271**	*FADS2*	A/T (0.89/0.11)	>0.80
**rs3834458**	*FADS2*	T/Z (0.70/0.30)	>0.90
**rs174448**	*FADS3*	T/C (0.69/0.31)	>0.90
**rs174449**	*FADS3*	T/C (0.69/0.31)	>0.90
**rs174455**	*FADS3*	T/C (0.68/0.32)	>0.90

FADS: Fatty Acid Desaturase; SNP: Single-Nucleotide polymorphism.

Supplemental Table 6. Pairwise comparison of predictive margins of the mixed effects logistic regression model.

	FO vs 5-MTHF	FO vs FO+5-MTFH	FO vs Placebo	5-MTFH vs FO+5-MTFH	5-MTFH vs Placebo	FO+5-MTFH vs Placebo
**7.5 years**						
CCTT-1 (sc)						
Contrast	-10.077	-5.546	-1.68	4.53	8.39	-3.86
95% CI	(-27.434, 7.29)	(-22.66, 11.57)	(-17.48,14.12)	(13.23, 23.30)	(-8.10, 24.89)	(-20.11,12.38)
p-value	0.255	0.526	0.835	0.617	0.319	0.641
STROOP Test– hits 1 (Word-reading)						
Contrast	1.05	-0.77	-2.32	-1.82	-3.38	1.55
95% CI	(-5.61, 7.72)	(-7.35, 5.80)	(-8.40, 3.75)	(-8.66, 5.009)	(-9.74, 2.97)	(-4.70,7.81)
p-value	0.756	0.818	0.453	0.600	0.291	0.627
STROOP Test – hits 2 (Color-naming)						
Contrast	0.37	4.44	1.09	4.07	0.72	3.35
95% CI	(-4.07, 4.81)	(0.06, 8.83)	(-2.95, 5.14)	(-0.47,8.62)	(-3.50, 4.95)	(-0.81,7.51)
p-value	0.870	**0.04**	0.595	0.07	0.737	0.115
SDMT – hits						
Contrast	-0.73	-0.97	1.64	-0.24	2.37	-2.61
95% CI	(-4.53,3.06)	(-4.72, 2.77)	(-1.81, 5.09)	(-4.13,6.64)	(-1.23,5.98)	(-6.17,0.93)
p-value	0.705	0.610	0.352	0.903	0.197	0.149
**9 years**						
CCTT-1 (sc)						
Contrast	-3.33	-28.99	-34.68	-25.65	-31.65	5.99
95% CI	(-34.89,28.19)	(-60.09, 2.11)	(-63.69,-6.28)	(-57.93, 6.62)	(-61.62, -1.68)	(-23.51,35.51)
p-value	0.756	**0.06**	**0.017**	0.119	**0.038**	0.69
STROOP Test – hits 1 (Word-reading)						
Contrast	-1.36	-0.13	1.03	1.49	2.39	-0.90
95% CI	(-8.22, 5.49)	(-6.86, 7.22)	(-5.37, 7.44)	(-5.56, 8.55)	(-4.07,8.87)	(-7.52, 5.71)
p-value	0.696	0.971	0.752	0.678	0.478	0.789
STROOP Test – hits 2 (Color-naming)						
Contrast	-0.95	2.56	1.68	3.52	2.63	0.88
95% CI	(-5.54, 3.66)	(-2.13,7.26)	(-2.61, 5.97)	(-1.20, 8.24)	(-1.68, 6.95)	(-3.55, 5.32)
p-value	0.683	0.285	0.443	0.144	0.231	0.696
SDMT – hits						
Contrast	-1.89	-0.45	3.72	1.43	5.61	-4.17
95% CI	(-5.87, 2.08)	(-4.48, 3.56)	(0.04, 7.39)	(-2.65, 5.52)	(1.87, 9.36)	(-7.97, -0.38)
p-value	0.350	0.823	**0.047**	0.491	**0.003**	0.031

5-MTHF: 5-Methyltetrahydrofolate; ***CCTT-1***: Children Color Trials Test; CI: confidence interval; FO: Fish-oil; sc: seconds; ***SDMT***: Symbol Digit Modalities Test. Bold: $p < 0.05$

Supplemental Table 7. Pairwise comparison of predictive margins of the mixed effects logistic regression model

	FO vs 5-MTHF	FO vs FO+5-MTFH	FO vs Placebo	5-MTFH vs FO+5-MTFH	5-MTFH vs Placebo	FO+5-MTFH vs Placebo
**7.5 years**						
**CCTT-1 (sc) p75**						
Contrast	0.06	0.21	0.23	0.14	0.16	-0.02
95% CI	(-0.12, 0.26)	(-0.002, 0.42)	(0.03, 0.42)	(-0.09, 0.37)	(-0.05, 0.38)	(-0.25, 0.25)
p-value	0.491	**0.05**	**0.018**	0.239	0.141	0.857
**STROOP Test – hits 1 (Word-reading) p50**						
Contrast	0.21	0.06	0.15	-0.14	-0.06	-0.08
95% CI	(-0.09, 0.52)	(-0.24, 0.37)	(-0.13, 0.44)	(-0.30, 0.33)	(-0.36, 0.24)	(-0.39, 0.22)
p-value	0.182	0.680	0.309	0.380	0.703	0.585
**STROOP Test – hits 2 (Color-naming) p75**						
Contrast	0.09	0.46	0.33	0.36	0.23	0.13
95% CI	(-0.38, 0.58)	(0.06, 0.85)	(-0.11, 0.78)	(-0.03, 0.43)	(-0.21, 0.67)	(-0.19, 0.67)
p-value	0.694	**0.023**	0.150	0.07	0.304	0.439
**SDMT - hits p50**						
Contrast	0.03	-0.02	0.16	-0.06	0.12	-0.19
95% CI	(-0.19, 0.26)	(-0.25, 0.20)	(-0.06, 0.39)	(-0.29, 0.17)	(-0.10, 0.36)	(-0.42, 0.03)
p-value	0.763	0.817	0.152	0.599	0.273	0.098
**9 years**						
**CCTT-1 (sc) p50**						
Contrast	-0.15	-0.25	0.03	-0.09	0.18	-0.28
95% CI	(-0.42, 0.10)	(-0.49, -0.007)	(-0.27, 0.29)	(-0.31, 0.12)	(-0.05, 0.43)	(-0.50, -0.06)
p-value	0.246	**0.043**	0.815	0.399	0.127	0.011
**STROOP Test – hits 1 (Word-reading) p75**						
Contrast	0.11	0.18	0.13	0.07	0.02	0.04
95% CI	(-0.20, 42)	(-0.12, 0.48)	(-0.16, 0.43)	(-0.26, 0.40)	(-0.29, 0.35)	(-0.26, 0.35)
p-value	0.495	0.246	0.367	0.676	0.875	0.780
**STROOP Test – hits 2 (Color-naming) p50**						
Contrast	-0.08	0.14	0.19	0.22	0.27	-0.04
95% CI	(-0.57, 0.40)	(-0.33, 0.61)	(-0.27, 0.65)	(-0.22, 0.67)	(-0.15, 0.71)	(-0.46, 0.36)
p-value	0.735	0.554	0.421	0.320	0.214	0.821
**SDMT - hits p75**						
Contrast	0.16	0.18	0.28	0.016	0.12	-0.105
95% CI	(-0.05, 0.39)	(-0.05, 0.42)	(0.07, 0.50)	(-0.24, 0.27)	(-0.12, 0.36)	(-0.36, 0.15)
p-value	0.147	0.132	**0.009**	0.903	0.327	0.418

5-MTHF: 5-Methyltetrahydrofolate***; CCTT-1***: Children Color Trials Test; CI: confidence interval; FO: Fish-oil; ***SDMT***: Symbol Digit Modalities Test. Bold: $p < 0.05$

Supplemental References

1. Koletzko, B.; Lattka, E.; Zeilinger, S.; Illig, T.; Steer, C. Genetic variants of the fatty acid desaturase gene cluster predict amounts of red blood cell docosahexaenoic and other polyunsaturated fatty acids in pregnant women: findings from the Avon Longitudinal Study of Parents and Children. *Am J ClinNutr* **2011**, *93(1),* 211-219, doi: 10.3945/ajcn.110.006189.
2. Lattka, E.; Koletzko, B.; Zeilinger, S.; Hibbeln, J.R.; Klopp, N.; Ring, S.M.; Steer, C. Umbilical cord PUFA are determined by maternal and child fatty acid desaturase *(FADS)* genetic variants in the Avon Longitudinal Study of Parents and Children (ALSPAC). *Br J Nutr* **2013**, *109(7)*, 1196-210, doi: 10.1017/S0007114512003108.
3. Chow, S.; Shao, J.; Wang, H. Sample size calculations in clinical research. *2nd Ed. Chapman & Hall/CRC Biostatistics Series*. **2008**, page 8

© 2020 by the authors. Licensee MDPI, Basel, Switzerland. This article is an open access article distributed under the terms and conditions of the Creative Commons Attribution (CC BY) license (http://creativecommons.org/licenses/by/4.0/).
